# SARS-CoV-2-infected cardiomyocytes exhibit upregulated necroptosis, but no evidence of mitochondrial permeability transition

**DOI:** 10.1016/j.jmccpl.2025.100833

**Published:** 2025-12-19

**Authors:** C. Gross, S. Chatterjee, B.E. Nilsson-Payant, S.D. Stojanović, H. Kefalakes, C. Bär, T. Thum, T. Pietschmann, J. Bauersachs, G. Amanakis

**Affiliations:** a Department of Cardiology and Angiology, Hannover Medical School, Hannover, Germany; b Institute of Molecular and Translational Therapeutic Strategies, Hannover Medical School, Hannover, Germany; c Fraunhofer Institute for Toxicology and Experimental Medicine ITEM, Hannover, Germany; d Fraunhofer Cluster of Excellence Immune-Mediated Diseases, Hannover, Germany; e Institute for Experimental Virology, TWINCORE Centre for Experimental and Clinical Infection Research, a joint venture between Hannover Medical School (MHH) and Helmholtz Centre for Infection Research (HZI), Hannover, Germany; f Cluster of Excellence RESIST (EXC 2155), Hannover Medical School, Hannover, Germany; g Department of Microbiology, Tumor and Cell Biology, Karolinska Institutet, Stockholm, Sweden; h Department of Gastroenterology, Hepatology, Infectious Diseases and Endocrinology, Hannover Medical School, Hannover, Germany; i German Center for Infection Research, Partner Site Hannover-Brunswick, Hannover, Germany; j PRACTIS Clinician Scientist Program, Dean's Office for Academic Career Development, Hannover Medical School, Hannover, Germany

**Keywords:** Cardiomyocytes, Induced pluripotent stem cells, SARS-CoV-2, COVID-19, Mitochondrial permeability transition, Necroptosis

## Abstract

Cardiac involvement in patients infected with COVID-19, in terms of myocarditis and troponin release, is associated with higher mortality. However, the underlying mechanisms are poorly understood.

Infection of cardiomyocytes derived from human induced pluripotent stem cells (iPSC-CMs) with a wild-type variant of SARS-CoV-2 exhibited a cardiotoxic effect. We examined whether elevated intramitochondrial calcium causes opening of the mitochondrial permeability transition pore (mPTP) leading to cell death. The mPTP inhibitor Cyclosporine A (CsA) did not improve viability, and phosphorylation levels of pyruvate dehydrogenase (PDH) remained similar pre- and post-infection, likely suggesting no substantial alteration of the intramitochondrial calcium level. Also, the protein expression of mitochondrial respiratory complexes did not change after SARS-CoV-2 infection.

Next, we examined whether cell death is related to necroptosis or pyroptosis upregulation. The phosphorylation level of receptor-interacting protein kinase 3 (RIP3) was elevated post-infection with SARS-CoV-2 while phosphorylation of mixed lineage kinase domain (MLKL)-S358 remained unaltered. This pattern may point toward an alternative regulation of necroptosis. Chemical inhibition of necroptosis (Necrostatin-1) and pyroptosis (MCC950) did not confer any protection. Notably, the phosphorylation of RIP3 under Necrostatin-1 was still elevated, suggesting that autophosphorylation of RIP3 may be a possible confounder.

Our data suggest that SARS-CoV-2 compromises cell viability in iPSC-CMs and may engage in non-canonical signaling via RIP3 phosphorylation. The lack of MLKL activation and the absence of protective effects from CsA indicate that neither classical necroptosis nor mitochondrial permeability transition are likely to be central regulators of cell death.

## Highlights

• Human iPSC-derived cardiomyocytes are permissive to SARS-CoV-2 infection.
• RIP3 phosphorylation increases post-infection, but not MLKL phosphorylation.
• Unaltered PDH phosphorylation and mitochondrial complex expression
• mPTP inhibition by Cyclosporine A fails to protect infected cardiomyocytes.

## Graphical abstract

**Figure:**
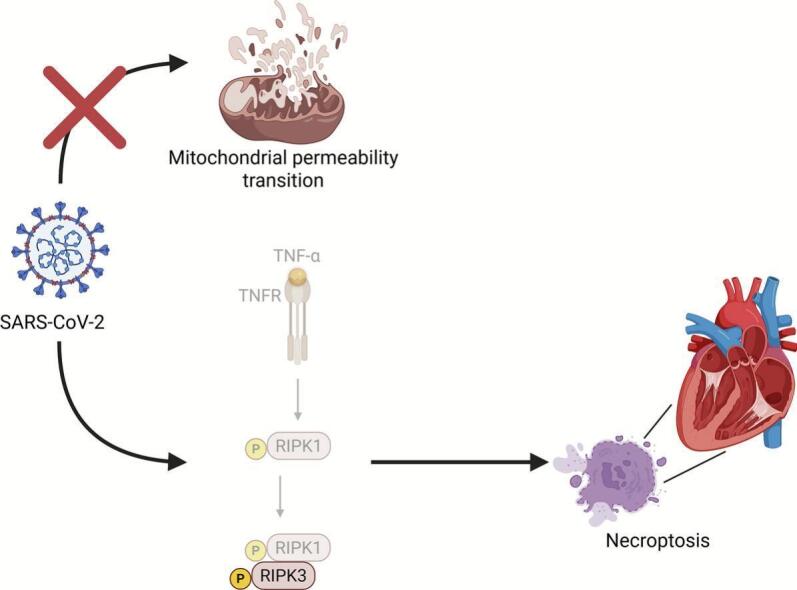

## 1 Introduction

The coronavirus disease 2019 (COVID-19) poses a significant health threat. COVID-19 shows an age-dependent phenotype, with elderly people and immunosuppressed individuals being particularly vulnerable [1]. While the primary clinical manifestations of COVID-19 involve the respiratory system, many of its symptoms such as fatigue and shortness-of-breath may also be attributed to the heart. Myocardial injury, as indicated by elevated cardiac biomarkers, has been observed in a substantial proportion of hospitalized patients and is associated with increased morbidity and mortality [2,3]. However, the precise mechanisms underlying COVID-19-related cardiomyopathy remain poorly understood.

Multiple cardiovascular abnormalities have been reported among patients with COVID-19 including myocardial inflammation, myocardial infarction, ventricular dysfunction as well as arrhythmias [4,5]. Mitochondria occupy 40 % of the volume of cardiomyocytes and produce 95 % of ATP that sustains heart contraction [6]. Thus, it is reasonable to assume that myocardial mitochondrial dysfunction plays a significant role in COVID-19. More recently, SARS-CoV-2 has been shown to alter cardiomyocyte physiology by inflicting mitochondrial damage in infected mice and humans [7].

In vitro models using human pluripotent stem cell-derived cardiomyocytes (iPSC-CMs) have provided critical insights into the permissiveness of these cells to SARS-CoV-2 infection and the ensuing cellular responses [[8], [9], [10], [11]]. Recent studies suggest that infected cardiomyocytes exhibit transcriptional, functional and morphological changes, including impaired contractility, endothelial damage, and cell death [[12], [13], [14]]. However, most of these studies relied mainly on transcriptomic analysis.

In the present manuscript, we take a hypothesis-oriented approach regarding the regulated forms of SARS-CoV-2-induced cell-death using an iPSC-CM model. We hypothesized that SARS-CoV-2 leads to increased intramitochondrial calcium activating the mitochondrial permeability transition pore and triggering apoptosis as the main mode of cell death in this model. Our findings aim to clarify the contribution of direct viral injury to COVID-19 cardiomyopathy and highlight potential therapeutic targets to mitigate cardiac complications in severe disease.

## 2 Material and methods

### 2.1 iPSCs culture and maintenance

Phoenix (hHSC_Iso4_ADCF_SeV-iPS2, alternative name: MHHi001-A) iPSC cells [15] were cultured in an incubator at 37 °C with 5 % CO_2_ on Geltrex-coated (Gibco, #A1413302) cell culture plates in E8 complete medium. The E8 media is home-made as described by Chen et al. [16] with the following ingredients: DMEM/F12, l-ascorbic acid-2-phosphate magnesium (64 mg/l), sodium selenium (14 μg/l), *E. coli* derived bFGF (20 μg/l), insulin (20 mg/l), NaHCO_3_ (543 mg/l) and holo-transferrin (10.7 mg/l), TGFβ1 (2 μg/l). Media was always adjusted to pH 7.4. The cells were passaged every five days with Versene (0.02 % EDTA in sterile phosphate-buffered saline (PBS) at pH 7.4) in E8 complete medium supplemented with 2 μM Thiazovivin (Selleckchem #S1459).

### 2.2 iPSC-CM differentiation and maintenance

iPSCs were seeded on Geltrex-coated 12-well cell culture plates and maintained and passaged in E8 complete medium. Differentiation was induced on day 0, when the iPSCs reached a confluency of about 70–80 %. To induce mesoderm specification, the medium was changed to Cardio Differentiation medium [RPMI 1640 + GlutaMAX + HEPES (Thermo Fisher Scientific, #72400021) supplemented with recombinant human albumin (Sigma-Aldrich, #A9731), l-Ascorbic acid 2-phosphate sesquimagnesium salt hydrate (LAA, Sigma-Aldrich, #A8960)] and the GSK3β inhibitor CHIR99021 (5 μM). On day 2 of differentiation Cardiac Differentiation medium was supplemented with 5 mM of the Wnt signaling inhibitor IWP2 (Peprotech, #S7085). Cardio Differentiation medium was changed every 48 h. On day 8, cells received Cardio Culture medium [RPMI 1640 + GlutaMAX + HEPES supplemented with 1 × B27 with insulin (Thermo Fisher Scientific, #17504-001)]. On day 9, wells with over 90 % iPSC-CM beating regions were selected to undergo massive CM expansion protocol [17]. The selected wells received Cardio Purification medium [RPMI 1640, no glucose, Gibco, #11879020] supplemented with 1 × B27 with insulin (Thermo Fisher Scientific, #17504-001) for 2–3 days (day 9–11 or 12). From day 11 onwards the differentiation wells were re-seeded on T175 or T75 at low cell density with Cardio Expansion medium changes every 2–3 days [Cardio culture medium + 3 μM GSK3β inhibitor CHIR99021]. Once the flask reached 100 % confluence, the expansion medium change was discontinued (around day 30–35 of differentiation). The expanded iPSC-CMs were purified once more using metabolic selection [18] by culturing the cells for 4–10 days in Cardio Selection medium [4 mM DL-lactate (Merck, #L4263, in 1 M HEPES, Carl Roth, #HN77.3), albumin, L-AA, in no glucose RPMI medium (RPMI 1640, no glucose, Gibco, #11879020)]. iPSC-CMs were maintained as described elsewhere [19]. The iPSC-CMs were re-plated on day 40–50 (calculated from day 0 of differentiation) into desired plate formats with Matrigel coating. All virus infection experiments with iPSC-CMs were performed day 60 onwards (calculated from day 0 of differentiation) using Cardio Culture medium in combination with presence/absence of inhibitors.

### 2.3 SARS-CoV-2 propagation and titration

SARS-CoV-2, isolate Munich-1.2/2020/984, was propagated and titrated as described previously [20,21]. In brief, African green monkey kidney Vero E6 cells (ATCC; CRL-1586) were infected with SARS-CoV-2 at a multiplicity of infection (MOI) of 0.001 in DMEM supplemented with 2 % fetal calf serum (FCS), MEM Non-Essential Amino Acids, 100 U/ml penicillin and 100 μg/ml streptomycin. At 3 days post-infection (dpi) virus-containing cell culture media was collected and cleared from cellular debris by centrifugation (2000 ×*g*, 5 min, 4 °C). Cleared viral stocks were subsequently concentrated and purified from small soluble protein contaminants such as cytokines that could adversely impact cells, using Amicon Ultra-15 Centrifugal Filter Units (100-kDa molecular weight cut-off) and reconstituted in PBS.

Infectious viral titers were determined by plaque assays in Vero E6 cells in minimum essential media (MEM) supplemented with 2 % FCS, 100 U/ml penicillin, 100 μg/ml streptomycin, 10 mM HEPES and MEM Non-Essential Amino Acids and 1.2 % Avicel CL-611. At 4 dpi semiliquid overlays were removed and cells were fixed in 4 % formaldehyde and plaques were visualized by staining with 1 % crystal violet.

All work involving live SARS-CoV-2 was performed in the biosafety level 3 facility of the Hannover Medical School in accordance with institutional biosafety requirements.

### 2.4 Treatment and infection of iPSC-CMs

iPSC-CMs were placed in the same media as during maintenance. Inhibition of the mitochondrial permeability transition pore opening (mPTP opening) was accomplished by addition of Cyclosporine A (CsA, 0.5 μM) 2 h prior to infection. Treatment with either Necrostatin-1 (Nec-1), a RIP kinase inhibitor (2.5 μM) or 0.05 μM MCC950, a nucleotide-binding oligomerization, leucine-rich repeat and pyrin domain-containing protein 3 (NLRP3) inhibitor (0.05 μM) was performed 16 h prior to infection. For iPSC-CM infection, cells were infected at an MOI of 0.1 (1 plaque forming unit per 10 cells). Following infection, cells remained at 37 °C with 5 % CO_2_ for 24 h before analysis.

### 2.5 Cytotoxicity assay (LDH)

Cytotoxicity was analyzed from Lactate Dehydrogenase (LDH) secreted into culture medium of infected and uninfected cells using the CyQUANT™ LDH Cytotoxicity Assay Kit (Invitrogen, #C20300) according to the manufacturer's instructions. Absorbance was measured at 490 nm and 680 nm on a Bio-Tek plate reader (Synergy HT).

### 2.6 RNA and real-time PCR

RNA was isolated with the QIAzol Lysis Reagent (QIAGEN, #79306) according to manufacturer's instructions. Concentration of the isolated RNA was measured using Take3 plates on a Bio-Tek plate reader (Synergy HT). RNA (500 ng) was reverse transcribed according to the manufacturer's instructions using the Takara PrimeScript RT Master Mix (#RR036A). A SYBR Green-based real-time PCR (40 cycles, Annealing: 30 s at 60 °C) was performed (Takara, #RR820A) on a CFX96 or CFX384 Real-Time System (Bio-Rad) using specific primer pairs. Average values from duplicates of each gene were used to calculate the relative abundance of transcripts, normalized to GAPDH and presented as fold change from 2^−ΔΔCT^. For detection of the housekeeping gene GAPDH, the following primers were used: F:5’-TTCACCACCATGGAGAAGGC-3’; R:5’-GGCATGGACTGTGGTCATGA-3’. For HPRT F:5’-AGGACTGAACGTCTTGCTCG-3’ and R:5’-GTCCCCTGTTGACTGGTCATT-3’ and for GUSB F:5’-GACACCCACCACCTACATCG-3’ and R:5’-CTTAAGTTGGCCCTGGGTCC-3’ were used. Viral Spike gene was amplified with the following primer pair: F:5’-CAACTGAAATCTATCAGGCCG-3’; R:5’-ACCAACACCATTAGTGGGTTG-3’. The following primers were used to amplify the genomic RNA for the inflammasome (NLRP3): F:5’-GAGGAAAAGGAAGGCCGACA-3’; R:5’-CAGGAGGAAGCACCTGGAAG-3’. For receptor-interacting protein kinase 3 (RIP3) the primer pair F:5’-ATCAGGGGGCTGAGAGACAA-3’ and R:5’-GTCTGGAGGAGGAGTCTGGT-3’ was used.

### 2.7 Immunofluorescence staining and microscopy

iPSC-CMs were seeded on Matrigel-coated cover slips and infected as described before; 24 h post-infection, the monolayer was washed twice with PBS and subsequently fixed with 4 % paraformaldehyde in PBS for 30 min at RT. The cells were incubated with quenching buffer (50 mM Na_4_Cl in PBS) for 5 min. Afterwards, the samples were blocked and permeabilized for 1–2 h at RT (0.3 % Triton X-100, 0.1 % BSA in PBS).

Primary antibodies were diluted in Dilution Buffer (0.3 % Triton X-100, 0.1 % BSA, 0.001 % Na-Azide in PBS) and incubated for 1–2 h at RT in the dark. The following antibodies were used in a 1:500 dilution: α-Actinin (Sigma, #A7811) and SARS-CoV-2 Nucleocapsid (SinoBiological, #40143-T62). Samples were rinsed in washing buffer (0.1 % BSA in PBS) three times, 3–5 min followed by incubation with the fluorescently labelled secondary antibody diluted 1:1000 in Dilution Buffer for 1–2 h at RT in the dark. Donkey α-mouse Cy3 IgG, (Jackson Immunoresearch, #715-165-150) and goat α-mouse IgG Alexa Fluor 647 (Thermo Fisher, #A21235) were used. Samples were rinsed in washing buffer three times, 3–5 min each followed by nuclei staining (1 drop in 500 μl dilution buffer, NuncBlue Live staining Ready Probes, Invitrogen, #R37605) for 15 min at RT in the dark. Images were acquired on a Nikon Eclipse Ni microscope (lenses: Apo Plan λ 20×, 0.75 NA OFN25 DIC N2 and Apo Plan λ 60×, 1.4 NA Oil, OFN25 DIC N2, camera: Andor DU-888 X-9999) and analyzed with ImageJ software version 1.54 f.

### 2.8 Quantification of infected iPSC-CM

From 1 differentiation round, iPSC-CMs were seeded on cover-slips coated with Matrigel in a 12 well plate format. 6 wells were infected, as normally with an MOI of 0.1 and incubated at 37 °C, 5 % CO2 for 24 h, while the other 6 wells were kept as uninfected controls. Following fixation and subsequent immune fluorescence staining, 10 random fields of view per sample were selected only by nuclear staining. Only α-actinin positive cells were taken into account for quantification. Nucleocapsid positive cells were divided by the number of the α-actinin positive cells.

### 2.9 TUNEL assay

From 1 differentiation round, iPSC-CMs were seeded on cover-slips coated with Matrigel in a 12 well plate format. 6 wells were infected, as normally with an MOI of 0.1 and incubated at 37 °C, 5 % CO2 for 24 h, while the other 6 wells were kept as uninfected controls. To assess the apoptosis rate in SARS-CoV-2 infected iPSC-CM a TUNEL assay (In Situ Cell Death Detection Kit, Fluorescein (Roche, #11684795910)) was performed. The monolayer was fixed for 30 min in 4 % PFA. After washing three times with PBS for 5 min at RT and subsequent quenching (50 mM Na_4_Cl in PBS), the TUNEL staining was performed according to manufacturers' instructions. To quantify the apoptosis rate, 10 randomly selected sample areas were imaged.

### 2.10 Protein isolation

The iPSC-CM monolayer was washed with PBS twice. PBS was aspirated and replaced with Lysis Buffer (Thermo Fisher Scientific, #J62289.AK) supplemented with Protease- und Phosphatase-Inhibitors (Thermo Scientific, #78440) and kept at RT for 10 min. The lysate was collected and stored at −80 °C.

Protein concentration was determined in a colorimetric microplate assay using a Pierce™ BCA Protein Assay (Thermo Fisher Scientific, #23227). Absorbance was measured at a wavelength of 562 nm on a Bio-Tek plate reader (Synergy HT).

### 2.11 Western blot analysis

Lysates were centrifuged at 11000 ×*g* for 10 min at 4 °C to remove cellular debris. Samples (20 μg protein) were analyzed on a 10 % SDS gel, run for 15 min at 70 V followed by 90 min at 100 V. Protein transfer was performed overnight at 4 °C at 30 V in Towbin Buffer (25 mM Tris, 192 mM glycine, 20 % methanol; pH 8.6) with 0.1 % SDS. The membrane was blocked for 1–2 h in 2 % BSA in TBST. The membrane was washed three times for 5 min in TBST followed by incubation of the primary antibody for 1–2 h at RT. The following primary antibodies were used at indicated concentrations: Spike protein (Thermo Fisher, #MA1-41173), RIP3-S227 (1:1000, Cell Signaling, #93654), RIP3 (1:500, Merck, #MABC1640), PDHA1 (1:1,000, Thermo Fisher Scientific, #45-6600); PDHA1-S293 (1:1000, Abcam, #ab177461), OXPHOS (1:250, Abcam, #ab110413), α-tubulin (1:5000, Cell Signaling, #2125S), cleaved caspase 3 (1:1000, Cell Signaling, #9661S), caspase 3 (1:1000, Cell Signaling, #9662S), MLKL S358 (1:000, Cell Signaling, #91689). After washing three times for 5 min in TBST, the secondary HRP-coupled antibody was added in a 1:10000 dilution and incubated for 1–2 h at RT. Secondary antibodies: anti-rabbit HRP (Cell Signalling, #7074S), anti-mouse HRP (Cell Signaling, #7076S) were used. The ECL substrate (Cytiva Amersham, #RPN2236) induced HRP chemiluminescence was visualized using the ChemiDoc Imaging System (BioRad) and quantified with ImageLab (BioRad, version 6.1). For reprobing, membranes were stripped with Restore™ PLUS Western Blot Stripping-Buffer (Thermo Fisher Scientific, #46430). Full western blots are presented in Suppl. Fig. 3C.

### 2.12 Statistics

Data presented was obtained from 11 differentiation rounds (iPSCs to cardiomyocytes). For each differentiation round, at least two separate wells of iPSC-CMs were allocated per experimental condition. Each data point in the figures represents one independent well. Data was analyzed with GraphPad Prism software (version 10.3) and are presented as mean ± SD. Outliers were identified using the ROUT method (Q = 0.5 %) and excluded from further analysis. Data sets were tested for normality of distribution using the Shapiro-Wilks test (threshold *P* < 0.05). For normally distributed data with only two groups, an unpaired student's *t*-test was conducted, while for ≥3 groups, a two-way-ANOVA was conducted with a post-hoc Tukey test for multiple comparisons. For non-normally distributed data a nonparametric test was used. Here, a Mann-Whitney test was performed for comparison of two groups and a Kruskal-Wallis test followed by a Dunn's multiple test comparison was used when comparing multiple groups (≥ 3). A *p*-value of ≥0.05 is considered non-significant, *p* < 0.05 = *, *p* < 0.01 = **, *p* < 0.001 = ***, *p* < 0.0001 = ****.

## 3 Results

### 3.1 Infection of iPSC-CMs by SARS-CoV-2 in vitro results in increased cellular cytotoxicity

Previous studies [22,23] have shown that cells transfected with spike-expressing pseudotyped particles of SARS-CoV-2 were able to infect various cell types, including cardiomyocytes, and result in altered electrophysiological behavior and structural damage of these cells. In this study we used the wild-type SARS-CoV-2 Munich isolate, the first emerging strain in Germany, to investigate the effects of SARS-CoV-2 infection on cardiomyocytes. iPSC-CMs were produced from a healthy donor iPSC line [15] which was recently validated as a model for SARS-CoV-2 infection [24] and used in this study after day 60 of differentiation.

**Fig. 1:**
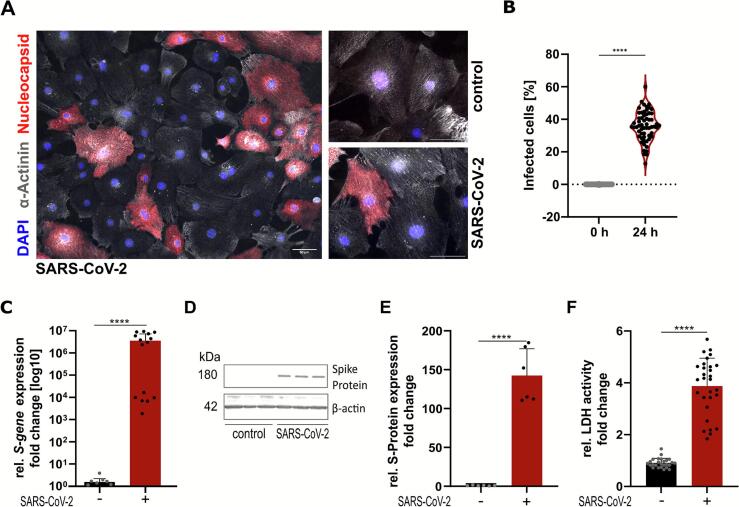
Infection of iPSC-CMs by SARS-CoV-2 in vitro results in increased cellular cytotoxicity. (A) Immunofluorescence staining shows infection of iPSC-derived cardiomyocytes. Nuclei (blue), α-Actinin (grey) and Nucleocapsid (red). Scale bar: 50 μm.(B) Quantification of infected cells at 24 h post-infection. (C) Infection of iPSC-derived cardiomyocytes is confirmed by expression of SARS-CoV-2 Spike RNA. (D) Western blot analysis confirms Spike Protein expression, quantified in (E). Full western blots in Suppl. Fig. 3A. (F) Cytotoxicity upon SARS-CoV-2 infection determined by activity of LDH released into the medium. Protein expression is normalized to β-actin, gene expression is normalized to GAPDH. *n =* 3 iPSC-CM differentiation rounds for all but (B), where *n* = 1 iPSC-CM differentiation round, statistical significance determined by Wilcoxon test (B), Kruskal-Wallis test (C, D), students *t*-test (F). *p* < 0.0001 = ****. Data are displayed as mean ± SD. (For interpretation of the references to color in this figure legend, the reader is referred to the web version of this article.) Infection of iPSC-CMs by SARS-CoV-2 in vitro results in increased cellular cytotoxicity. (A) Immunofluorescence staining shows infection of iPSC-derived cardiomyocytes. Nuclei (blue), α-Actinin (grey) and Nucleocapsid (red). Scale bar: 50 μm.(B) Quantification of infected cells at 24 h post-infection. (C) Infection of iPSC-derived cardiomyocytes is confirmed by expression of SARS-CoV-2 Spike RNA. (D) Western blot analysis confirms Spike Protein expression, quantified in (E). Full western blots in Suppl. Fig. 3A. (F) Cytotoxicity upon SARS-CoV-2 infection determined by activity of LDH released into the medium. Protein expression is normalized to β-actin, gene expression is normalized to GAPDH. *n =* 3 iPSC-CM differentiation rounds for all but (B), where *n* = 1 iPSC-CM differentiation round, statistical significance determined by Wilcoxon test (B), Kruskal-Wallis test (C, D), students *t*-test (F). *p* < 0.0001 = ****. Data are displayed as mean ± SD. (For interpretation of the references to color in this figure legend, the reader is referred to the web version of this article.)

Immunofluorescence staining of fixed cells post-infection (Fig. 1A) confirmed successful SARS-CoV-2 infection in iPSC-CMs. Staining against SARS-CoV-2 nucleocapsid results in robust staining of infected cells. iPSC-CM were infected with an MOI of 0.1. After 24 h of incubation, we observed an increase of infected cells to ∼35 % (Fig. 1B).

Infection of iPSC-CMs was further confirmed by detection of viral RNA by RT-qPCR (Fig. 1C) and Spike protein level in cell lysates by western blot analysis (Fig. 1D, E). Overall, after 24 h post-infection with the SARS-CoV-2 Munich isolate was accompanied by increased cell death as measured by secretion of LDH into the culture medium. Specifically, infection resulted in a threefold change in cytotoxicity compared to the uninfected control (Fig. 1F).

Overall, the data demonstrates that iPSC-CMs can be directly infected by the SARS-CoV-2 Munich isolate.

### 3.2 PDH phosphorylation level is unaffected following SARS-CoV-2 infection

**Fig. 2:**
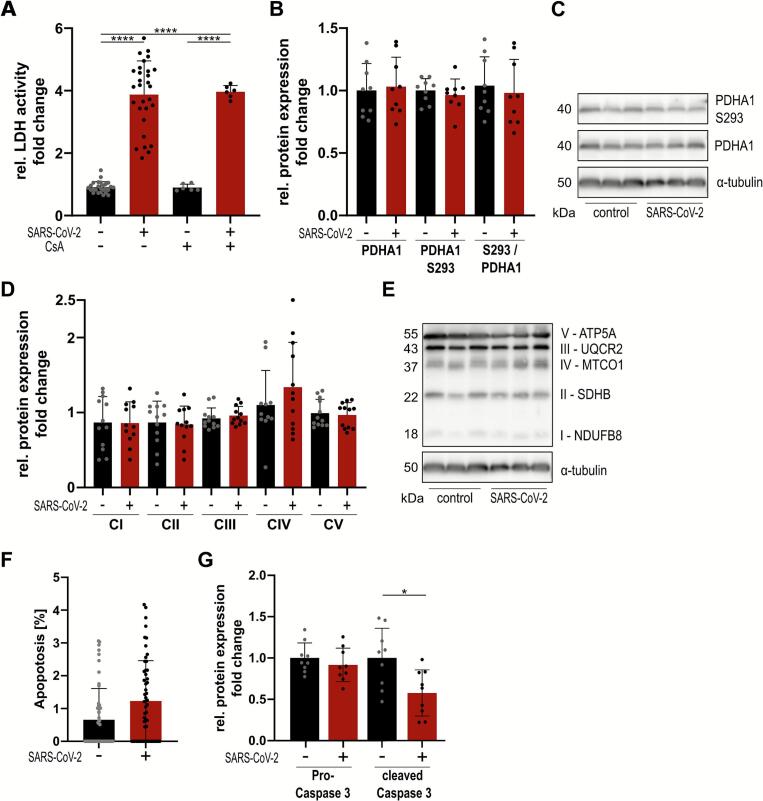
PDH phosphorylation is unaffected following SARS-CoV2 infection. (A) Cytotoxicity after Cyclosporine A (CsA) treatment [0.5 μM, 2 h] as determined by activity of LDH released into the medium. (B) Quantification of pyruvate dehydrogenase A1 (PDHA1) protein expression and its phosphorylation on serine 293. Values normalized to α-tubulin. (C) Representative Western Blot. (D) Quantification of CI = Complex I, CII = Complex II, CIII = Complex III, CIV = Complex IV and CV = Complex V normalized to α-tubulin. Full western blots in Suppl. Fig. 3B. (E) Protein expression of the respiratory chain following SARS-CoV-2 infection. Full western blots in Suppl. Fig. 3B. (F) Apoptosis rate determined by TUNEL staining 24 h post-infection with an MOI of 0.1. (G) Quantification of procaspase 3 and cleaved caspase 3 protein expression. Values normalized to α-tubulin. Corresponding western blots in Suppl. Fig. 3C. *n* = 3 iPSC-CM differentiation rounds for all but (A, B), where *n* = 1 iPSC-CM differentiation round. Statistical significance determined by two-way ANOVA (A, B), Mann-Whitney test (F) or t-test (D, G). *p* ≤ 0.05 = *, p < 0.0001 = ****. Data are displayed as mean ± SD. PDH phosphorylation is unaffected following SARS-CoV2 infection. (A) Cytotoxicity after Cyclosporine A (CsA) treatment [0.5 μM, 2 h] as determined by activity of LDH released into the medium. (B) Quantification of pyruvate dehydrogenase A1 (PDHA1) protein expression and its phosphorylation on serine 293. Values normalized to α-tubulin. (C) Representative Western Blot. (D) Quantification of CI = Complex I, CII = Complex II, CIII = Complex III, CIV = Complex IV and CV = Complex V normalized to α-tubulin. Full western blots in Suppl. Fig. 3B. (E) Protein expression of the respiratory chain following SARS-CoV-2 infection. Full western blots in Suppl. Fig. 3B. (F) Apoptosis rate determined by TUNEL staining 24 h post-infection with an MOI of 0.1. (G) Quantification of procaspase 3 and cleaved caspase 3 protein expression. Values normalized to α-tubulin. Corresponding western blots in Suppl. Fig. 3C. *n* = 3 iPSC-CM differentiation rounds for all but (A, B), where *n* = 1 iPSC-CM differentiation round. Statistical significance determined by two-way ANOVA (A, B), Mann-Whitney test (F) or t-test (D, G). *p* ≤ 0.05 = *, p < 0.0001 = ****. Data are displayed as mean ± SD.

SARS-CoV-2 infection affects pathways involved in endoplasmic-reticulum stress [25]. To address whether myocardial cell death is a result of increased intramitochondrial calcium, and the activation of the mitochondrial permeability transition pore (mPTP), we treated iPSC-CMs with Cyclosporine A (CsA- 0.5 μM) 2 h prior to infection. CsA desensitizes the mPTP by binding to and inhibiting the ability of Cyclophilin D to activate the mPTP [26]. To our surprise, while SARS-CoV-2 infection increased lactate dehydrogenase (LDH) release, a marker of cytotoxicity, CsA pretreatment did not alter LDH release upon SARS-CoV-2 infection, suggesting no differences in cytotoxicity (Fig. 2A).

To further elucidate whether the intramitochondrial calcium concentration is affected by SARS-CoV-2, we measured the phosphorylation of pyruvate dehydrogenase (PDH) in protein lysates from uninfected and infected cardiomyocytes. We found the level of total and phosphorylated (inactive) PDH unaltered following SARS-CoV-2 infection (Fig. 2B, C). We also measured the expression of respiratory chain complexes by western blot analysis (Fig. 2D, E). Interestingly, no significant changes in all five complexes were observed. This suggests that SARS-CoV-2 infection does not affect the expression level of the mitochondrial respiratory complexes in this model.

To examine if apoptosis plays a major role in SARS-CoV-2 infected iPSC-CM, we performed TUNEL staining (Fig. 2F) on fixed cells 24 h post-infection and also checked for expression of procaspase 3 and cleaved caspase 3 in protein lysates from uninfected and infected samples (Fig. 2G). No significant differences were observed in either TUNEL positive cells or expression of procaspase 3. However, we observed a significant decrease in cleaved caspase 3 following infection, suggesting that apoptosis is not a main mechanism of SARS-CoV2-mediated cardiotoxicity.

### 3.3 SARS-CoV-2 infection leads to upregulation of necroptosis

The previous results suggest that upregulation of an alternate pathway is responsible for myocardial cell death in this model. We hypothesized that necroptosis is upregulated after SARS-CoV-2 infection.

**Fig. 3:**
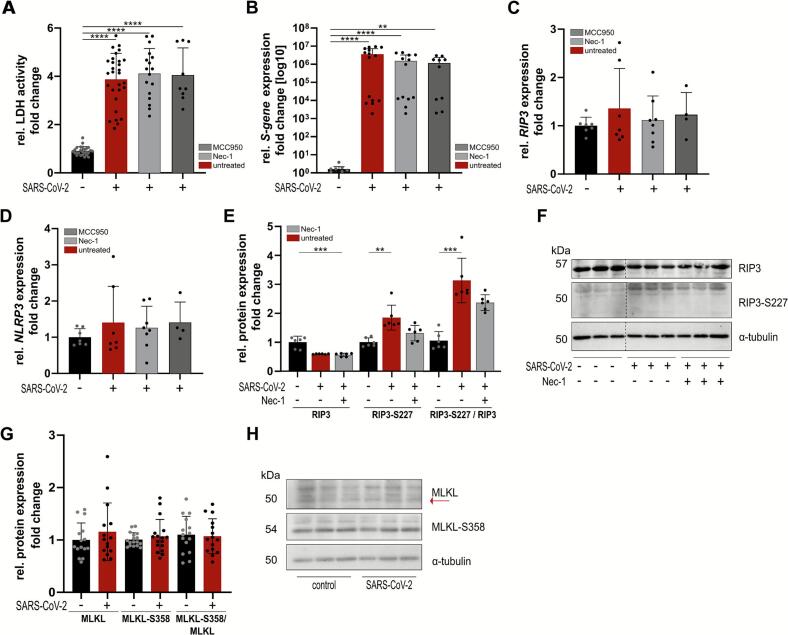
Increased necroptosis following SARS-CoV-2 infection. (A) Cytotoxicity after Necrostatin-1 (Nec-1) [2.5 μM] or MCC950 [0.05 μM] treatment as determined by activity of LDH released into the medium. (B) Spike- (C) RIP3 and (D) NLR family pyrin domain containing 3 (NLRP3) inflammasome gene expression following SARS-CoV-2 infection. Gene expression normalized to GAPDH. (E) Quantification of RIP3 and RIP3-S227 protein expression after Nec-1 treatment. (F) Representative WB for RIP3 and RIP3-S227 following SARS-CoV-2 infection. Full western blots in Suppl. Fig. 3D. (G) Quantification of MLKL and MLKL-S358 protein expression with a representative WB (H). Full western blots in Suppl. Fig. 3E. *n* = 3 iPSC-CM differentiation rounds, statistical significance determined by two-way ANOVA (A) or Kruskal-Wallis test (B–E) or Mann-Whitney (G). *p* < 0.01 = **, p < 0.0001 = ****. Data are displayed as mean ± SD. Increased necroptosis following SARS-CoV-2 infection. (A) Cytotoxicity after Necrostatin-1 (Nec-1) [2.5 μM] or MCC950 [0.05 μM] treatment as determined by activity of LDH released into the medium. (B) Spike- (C) RIP3 and (D) NLR family pyrin domain containing 3 (NLRP3) inflammasome gene expression following SARS-CoV-2 infection. Gene expression normalized to GAPDH. (E) Quantification of RIP3 and RIP3-S227 protein expression after Nec-1 treatment. (F) Representative WB for RIP3 and RIP3-S227 following SARS-CoV-2 infection. Full western blots in Suppl. Fig. 3D. (G) Quantification of MLKL and MLKL-S358 protein expression with a representative WB (H). Full western blots in Suppl. Fig. 3E. *n* = 3 iPSC-CM differentiation rounds, statistical significance determined by two-way ANOVA (A) or Kruskal-Wallis test (B–E) or Mann-Whitney (G). *p* < 0.01 = **, p < 0.0001 = ****. Data are displayed as mean ± SD.

Since the receptor-interacting protein kinase 1 (RIP1) has been described as an essential component for RIP3 activation during necroptosis [25], we treated iPSC-CMs with 2.5 μM Necrostatin-1 (Nec-1), an inhibitor of the RIP1 kinase that prevents its phosphorylation and activation. Another cell-death pathway prominent in peripheral blood mononuclear cells in patients with COVID-19 is pyroptosis, characterized by NLRP3 activation [27]. To this extent, cardiomyocytes were treated with 0.05 μM MCC950, a potent and specific inhibitor of the NLRP3 inflammasome. Treatment with either inhibitor (Nec-1 or MCC950) did not lead to increased cytotoxicity in iPSC-CMs, as demonstrated by similar LDH release in the supernatant of either MCC950 or Nec-1 treated cell cultures compared to the untreated control, showing that none of these inhibitors can protect iPSC-CMs from myocardial cell death following SARS-CoV-2 infection (Fig. 3A).

We next examined whether viral replication is affected by RIP kinase or NLRP3 inflammasome inhibition by RT-qPCR for the SARS-CoV-2 spike gene. However, treatment with MCC950 or Nec-1 did not alter the presence of the SARS-CoV-2 spike RNA compared to the untreated control (Fig. 3B). Although the inhibitors act at the protein level, we aimed to exclude potential transcriptional effects caused by SARS-CoV-2 infection itself. To this end, we quantified mRNA expression of RIP3 (Fig. 3C) and NLRP3 (Fig. 3D) by RT-qPCR. They revealed similar expression following SARS-CoV-2 infection and pre-treatment with cell death pathway inhibitors compared to untreated samples. Next, we investigated whether infection of iPSC-CM affects RIP3 phosphorylation. Surprisingly, we observed no changes in total RIP3 protein expression but a significant increase of RIP3 phosphorylation on serine 227 in iPSC-CM samples post infection (Fig. 3E, F). In line with these findings, treatment of iPSC-CMs with Nec-1 prior to infection results in unaltered RIP3 expression, whereas phosphorylation on serine 227 was increased compared to uninfected samples, but to a lesser extent than untreated samples with Nec-1. Next, we addressed, whether downstream signaling of RIP3 is also altered following SARS-CoV-2 infection. We therefore analyzed protein expression of MLKL and its phosphorylation on S358. Interestingly, we observed no changes in protein expression following infection.

## 4 Discussion

In this study, we provide evidence that SARS-CoV-2 infection affects cardiomyocyte viability in-vitro. iPSC-CMs exhibit upregulated necroptosis upon infection. Our iPSC-CM model demonstrated that SARS-CoV-2 can directly trigger cell death pathways in human cardiomyocytes, notably via RIP3 phosphorylation, indicating necroptosis. CsA failed to exert a protective effect, indicating that mitochondrial permeability transition is unlikely to play a central role in cell death regulation within this model. Moreover, phosphorylation levels of PDHA at serine 293 remained unchanged following SARS-CoV-2 infection, likely suggesting that intramitochondrial calcium levels are similar prior to and after SARS-CoV-2 infection.

Early on during the COVID-19 pandemic it was shown that SARS-CoV-2 infects cardiomyocytes in vitro through the ACE2-receptor [8]. RNA sequencing demonstrated that infected iPSC-CMs respond strongly to viral infection at the transcriptional level including interferon activation and signatures of apoptosis (measured by cleaved caspase 3) and oxidative stress [8]. In addition, SARS-CoV-2 infection deregulates pathways involved in endoplasmic-reticulum stress [28,29] and protein homeostasis [25]. The ER is a major source of calcium in cardiomyocytes since it plays a key role in modulating their contractility [30]. This led us to question whether SARS-CoV-2 induces the release of calcium by the ER, which subsequently activates mitochondrial permeability transition and triggers apoptotic cell death in this model. Measuring intramitochondrial calcium in a biosafety level 3 setting using chemical or genetic calcium probes was challenging so we opted for a surrogate – the phosphorylation level of PDHA1, as it is inversely regulated by the mitochondrial calcium concentration [31]. We also employed an inhibitor of mitochondrial permeability transition – CsA at a lower dose than required for immunomodulation through calcineurin inhibition [26]. In our model, SARS-CoV-2 infection does not affect the level of PDHA1 phosphorylation, and CsA does not confer cardioprotection. The protein expression level of respiratory complexes did not differ in infected versus uninfected cardiomyocytes. Notably, there is considerable variability in published data regarding the respiratory complexes; heart-tissue RNAseq data from C57BL6 mice infected with SARS-CoV-2 exhibit less mRNA of electron transport chain complexes compared to untreated mice [32], whereas in SARS-CoV-2 infected HEK293 cells an increased protein expression level of most respiratory complexes was seen [11,33,34]. These observations may be caused by differential activation of canonical negative feedback mechanisms, which could be operative on certain experimental settings but absent or suppressed in others. Our data indicates calcium dependent regulation of PDH remains stable prior and after SARS-CoV-2 infection suggesting intramitochondrial calcium levels are similar prior to and after SARS-CoV-2 infection, although the data does not directly evaluate this but future research in this area is warranted. Of note, in HeLa cells it has been shown that SARS-CoV-2 infection affects the intracellular calcium homeostasis [29]. It should also be noted that SARS-CoV-2 infection may increase mitochondrial calcium levels, leading to PDH dephosphorylation. However, a simultaneous upregulation of PDH kinases could possibly counteract this effect, resulting in no apparent change in the phosphorylation status of PDH.

At this point, we considered two other cell-death pathways that have been implicated in SARS-CoV-2 mediated cell-death. Pyroptosis is a cell-death mode where NLRP3 plays a central role. The protein 3a of SARS-CoV-2 can activate NLRP3 in macrophages in patients with COVID-19 complicated by ARDS [35]. Furthermore, post-mortem analysis of lung and heart tissue from COVID-19 patients revealed elevated caspase-1 expression, indicating activation of the pyroptotic cell death pathway and suggesting a role for inflammasome-driven inflammation in disease pathology [36]. We hypothesized that direct inhibition of NLRP3 activation results in protection of cardiomyocytes in SARS-CoV-2 infection. MCC950 targets the NLRP3 protein by binding to it on a domain with ATPase activity, crucial for its oligomerization and subsequent activation [37]. However, direct pharmacological inhibition of NLRP3 activation using MCC950 did not attenuate cell death.

Another cell-death mode we considered was necroptosis. Necroptosis is activated by the TNF-alpha receptor and involves the receptor-interacting protein kinases 1 and 3 (RIP1, RIP3) as well as the mixed lineage kinase domain-like proteins (MLKL) [38]. It has been shown that RIP3 leads to the oligomerization of SARS-CoV-2 protein 3a in lung cells promoting cell-death [39]. Other clinical studies have shown that patients with acute respiratory distress syndrome exhibit a higher RIP3 level in serum [40]. Thus, we hypothesized that necroptosis is the main pathway via which cell-death occurs in cardiomyocytes. To this extent, we looked at RIP3 phosphorylation. While total RIP3 expression was not affected, the cardiomyocytes exhibited a strong increase in RIP3 phosphorylation 24 h post-infection, while MLKL phosphorylation remained unaltered. This suggests that although RIP3 is activated, the canonical pathway of necroptotic cell death via MLKL is not engaged in this cellular context. Interestingly, MLKL-independent necroptosis has been described in other systems and may represent a non-canonical form of regulated necrosis [41]. Next, we employed Nec-1, an inhibitor of RIP1 phosphorylation and of the RIP1/RIP3/MLKL signal transduction. To our surprise however, Nec-1 did not attenuate cell-death nor reduce RIP3 phosphorylation to baseline levels. These findings are in line with studies reporting that RIP3 can undergo autophosphorylation, which drives necroptosis independent of RIP1 [42]. Moreover, RIP1 has been shown to act as a suppressor of RIP3-mediated necroptosis under certain conditions. This very inhibition may paradoxically enhance RIP3 activity [43].

Since RIP3 is a kinase, further research is warranted in order to assess whether it targets PDH in similar models. It has been demonstrated that RIP3 directly phosphorylates the E3 subunit of PDH at threonine 135 [44]. This phosphorylation activates PDH, enhancing aerobic respiration and increasing mitochondrial reactive oxygen species production during TNF-induced necroptosis. Interestingly, the study also found that MLKL, another necroptosis effector, is required for RIP3 to translocate and interact with mitochondria-localized PDH, suggesting a coordinated mechanism in necroptotic signaling and metabolic regulation.

We are well aware, that iPSC-CMs exhibit properties that differ substantially from those of adult human cardiomyocytes. All experiments in the present study were performed after 60 days of differentiation, when the cells majorly rely on oxidative phosphorylation but still have more scope for further metabolic maturation when compared to an adult human cardiomyocyte. Additionally, in the intact heart cardiomyocytes are embedded within a complex and compact multicellular tissue architecture, not represented by monolayer cultures. Infection of other cardiac cell types such as the coronary vasculature or immune cells present in the intact heart play a significant role in the cardiac complications observed in patients with COVID-19 [45], as processes such as endothelial dysfunction, formation of microthrombi or immune cell infiltration have synergistic effects that, of course, cannot be replicated in iPSC-CMs alone. It is worth noting that all inhibitors in the present study (Nec-1, MCC950 and CsA) lacked any protective effect. Other confounders regarding this outcome may possibly be insufficient inhibitor uptake, as in vitro conditions may not replicate pharmacokinetics and bioavailability from in vivo settings. Furthermore, metabolic inactivation, instability or off-target effects may contribute as distorting factors.

The combined data in the present study support direct cardiac injury in COVID-19. Direct viral cytotoxicity through necroptosis may be relevant in cases where the immune system fails to control the disease. This is in line with previous studies showing inflammasome activation in peripheral blood cells of severely ill COVID-19 patients. Our results suggest that targeting necroptosis may represent a therapeutic strategy, warranting further investigation into both pharmacological and genetic approaches to modulate this pathway in SARS-CoV-2 infection.

The following are the supplementary data related to this article.

**Supplementary Fig. 1:**
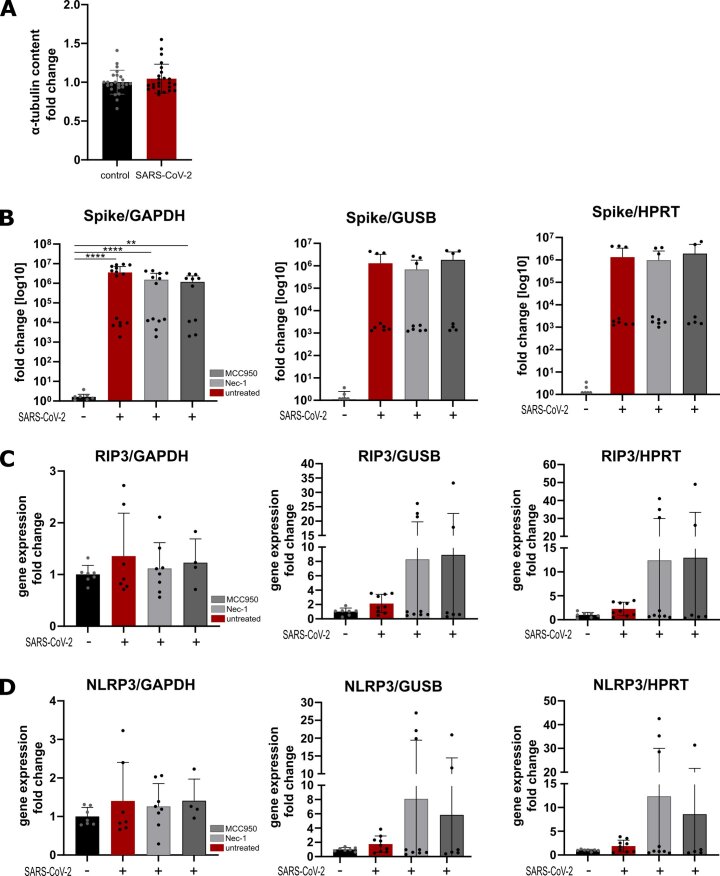
Internal controls and housekeeping genes. (A) Comparison of α-tubulin protein expression throughout all performed western blots from uninfected and SARS-CoV-2 infected samples. (B) Relative gene expression of SARS-CoV-2 Spike against GAPDH (left), HPRT (middle) and GUSB (right). (C) relative gene expression of RIP3 against GAPDH (left), HPRT (middle) and GUSB (right). (D) Relative gene expression of NLRP3 against GAPDH (left), HPRT (middle) and GUSB (right). *n* = 3 iPSC-CM differentiation rounds, statistical significance determined by students *t*-test (A) or Kruskal-Wallis test (B–D). Data are displayed as mean ± SD. *p*-value of ≥0.05 = non-significant.

**Supplementary Fig. 2:**
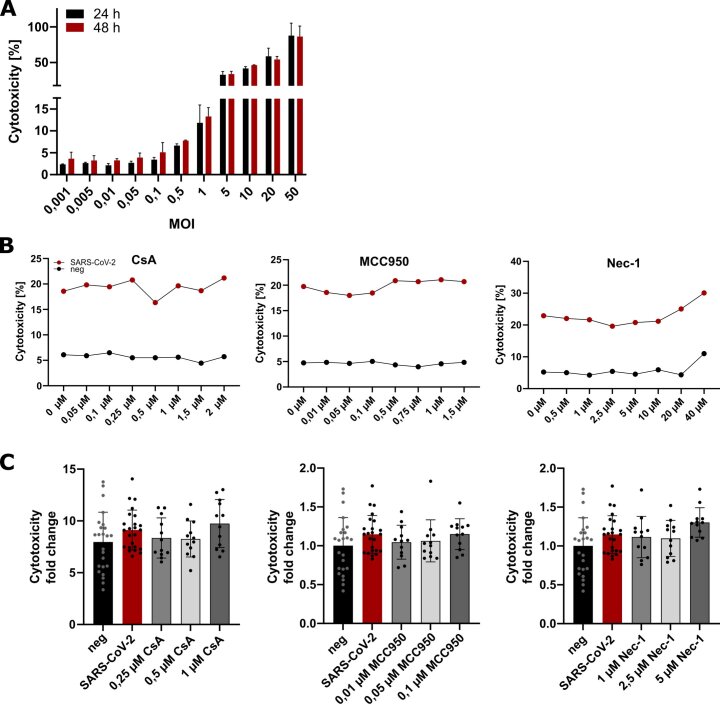
SARS-CoV-2 and inhibitor titration. (A) Cytotoxicity upon infection determined by activity of LDH released into the medium. MOIs from 0,001 to 50 were used to determine cytotoxicity after 24 h (black bars) and 48 h (red bars). (B) Cytotoxicity measurements from titration of Cyclosporine A (left), MCC950 (middle) and Necrostatin-1 (right). (C) Cytotoxicity measurements of smaller range of compounds. CsA (left), MCC950 (middle) and Nec-1 (right). *n* = 2 iPSC-CM differentiation rounds, statistical significance determined by students t-test between (A, B) or Kruskal-Wallis test (C). Data are displayed as mean ± SD. p-value of ≥0.05 = non-significant.

**Supplementary Fig. 3:**
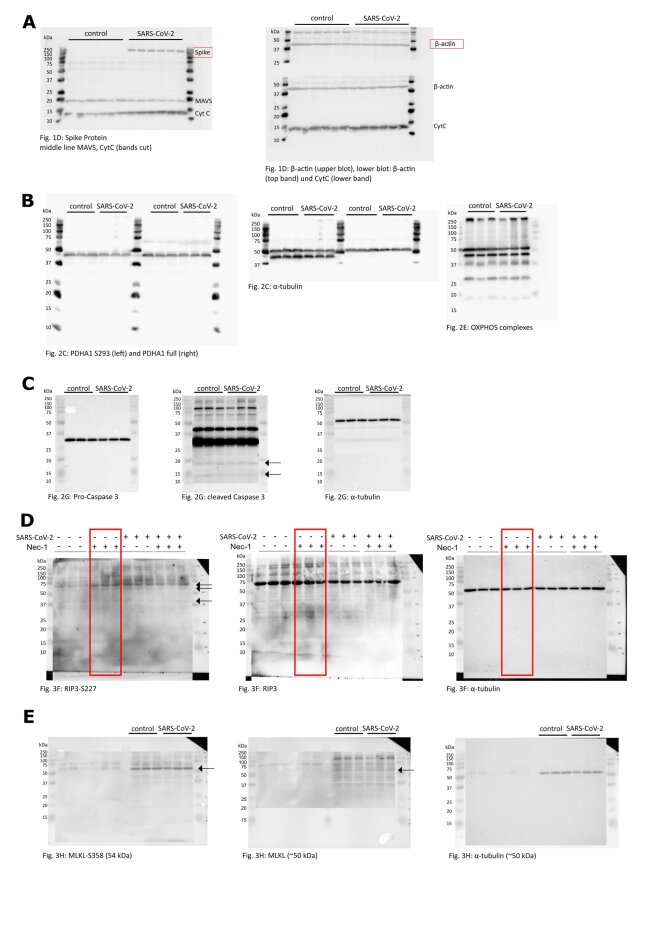
Representative uncut western blots. (A) Full Blots belonging to Fig. 1. Membrane was cut horizontally and incubated with different antibodies and assembled for detection. (B) Full blots from Fig. 2C. Left: The membrane was cut vertically in the middle. The left part was incubated with PDHA1-S227 antibody and the right half with PDHA1. After stripping, both halves were reprobed with α-tubulin (middle) and the right part was reprobed, again, for OXPHOS complexes (right). (C) Full blots for quantification of procaspase 3 (left) and cleaved caspase 3 (middle) in Fig. 2G, α-tubulin as loading control (right). (D) Full blots for Fig. 3F. Left: RIP3-S227, middle: RIP3 and right: α-tubulin. The red box denotes irrelevant conditions not included in Fig. 3F. (E) Displays the full Blot from Fig. 3H. Left: MLKL-S358, middle: after stripping and reprobing MLKL and α-tubulin on the right, after another stripping and reprobing.

## CRediT authorship contribution statement

**C. Gross:** Writing – review & editing, Writing – original draft, Visualization, Validation, Project administration, Methodology, Investigation, Formal analysis, Data curation, Conceptualization. **S. Chatterjee:** Resources, Methodology, Formal analysis. **B.E. Nilsson-Payant:** Resources, Methodology, Formal analysis. **S.D. Stojanović:** Formal analysis. **H. Kefalakes:** Formal analysis. **C. Bär:** Formal analysis. **T. Thum:** Resources, Methodology, Funding acquisition, Formal analysis. **T. Pietschmann:** Supervision, Resources, Methodology, Funding acquisition, Formal analysis. **J. Bauersachs:** Writing – review & editing, Supervision, Resources, Funding acquisition, Formal analysis. **G. Amanakis:** Writing – review & editing, Writing – original draft, Validation, Supervision, Resources, Project administration, Methodology, Investigation, Funding acquisition, Formal analysis, Data curation, Conceptualization.

## Funding

This research project was supported by the PRACTIS Clinician Scientist Program, funded by 10.13039/501100005624Hannover Medical School (MHH) and the 10.13039/501100001659Deutsche Forschungsgemeinschaft (DFG, German Research Foundation) - DFG ME 3696/3, and by the internal funding program (HiLF) of 10.13039/501100005624Hannover Medical School, both to GA. This study was supported by the COVID-19 Research Network Lower Saxony (COFONI) with funding from the Ministry of Science and Culture of Lower Saxony, Germany (14-76403-184) (3F22 to TT). This publication was supported by the European Virus Archive GLOBAL (EVA-GLOBAL) project that has received funding from the 10.13039/501100000780European Union's 10.13039/100010661Horizon 2020 - Research and Innovation Framework Programme under grant agreement No. 871029. This study was also funded by the 10.13039/501100001659Deutsche Forschungsgemeinschaft (DFG, German Research Foundation) under Germany's Excellence Strategy - EXC 2155 - project number 390874280 (to BNP, HK and TP).

## Declaration of competing interest

TT is founder and CSO/CMO of Cardior Pharmaceuticals, a wholly-owned subsidiary of Novo Nordisk (outside of this study). TT is board member of RNATICS GmbH (outside of this study).

No related conflicts of interest declared by the authors with respect to the content of the presented work.
